# C-KIT Signaling Depends on Microphthalmia-Associated Transcription Factor for Effects on Cell Proliferation

**DOI:** 10.1371/journal.pone.0024064

**Published:** 2011-08-24

**Authors:** Bengt Phung, Jianmin Sun, Alexander Schepsky, Eirikur Steingrimsson, Lars Rönnstrand

**Affiliations:** 1 Wallenberg Laboratory, Experimental Clinical Chemistry, Department of Laboratory Medicine, Lund University, Skåne University Hospital, Malmö, Sweden; 2 Department of Biochemistry and Molecular Biology, Faculty of Medicine, University of Iceland, Reykjavík, Iceland; Istituto Dermopatico dell'Immacolata, Italy

## Abstract

The development of melanocytes is regulated by the tyrosine kinase receptor c-KIT and the basic-helix-loop-helix-leucine zipper transcription factor Mitf. These essential melanocyte survival regulators are also well known oncogenic factors in malignant melanoma. Despite their importance, not much is known about the regulatory mechanisms and signaling pathways involved. In this study, we therefore sought to identify the signaling pathways and mechanisms involved in c-KIT mediated regulation of Mitf. We report that c-KIT stimulation leads to the activation of Mitf specifically through the c-KIT phosphorylation sites Y721 (PI3 kinase binding site), Y568 and Y570 (Src binding site). Our study not only confirms the involvement of Ras-Erk signaling pathway in the activation of Mitf, but also establishes that Src kinase binding to Y568 and Y570 of c-KIT is required. Using specific inhibitors we observe and verify that c-KIT induced activation of Mitf is dependent on PI3-, Akt-, Src-, p38- or Mek kinases. Moreover, the proliferative effect of c-KIT is dependent on Mitf in HEK293T cells. In contrast, c-KIT Y568F and Y721F mutants are less effective in driving cell proliferation, compared to wild type c-KIT. Our results reveal novel mechanisms by which c-KIT signaling regulates Mitf, with implications for understanding both melanocyte development and melanoma.

## Introduction

Cell signaling plays an important role in the fine tuning of cellular function and behavior. Signaling cascades generated by the cell surface tyrosine kinase receptor c-KIT through the binding of its ligand stem cell factor (SCF) are involved in the regulation of many cell types including melanocytes [1], mast cells [2], [3], germ cells [4] and interstitial cells of Cajal [5]. Loss-of-function mutations of the receptor or its ligand lead to abnormalities in pigmentation [6]–[8], hematopoiesis [9], [10], gametogenesis [11], [12] and gut motility [13]. The binding of SCF to c-KIT leads to dimerization and auto-phosphorylation of tyrosine residues located in the intracellular part of the receptor. Phosphorylation of tyrosine residues enables c-KIT to recruit and bind to downstream signaling proteins for subsequent activation of signal transduction pathways. It is well characterized that the c-KIT phosphorylation sites Y568 and Y570 can act as docking and activation sites for Src family kinases. The transduction signal relayed from c-KIT to Src kinases triggers the activation of the Ras-Erk pathway, involving the pro-survival and anti-apoptotic Ras-Raf-Mek-Erk cascade [14], [15]. The activation of Src kinase also regulates the stress-activated protein kinase p38 [16]. On the other hand, the phosphatidylinositide 3 kinase (PI3 kinase) survival pathway is switched on by phosphorylation of c-KIT at Y721. The activation can either be set in motion by the direct binding of the p85 subunit of PI3 kinase to Y721 or by binding of PI3 kinase to the scaffolding protein Grb2 associated binding protein (Gab2). Grb2 that is bound to phosphorylated Y703 and Y936 in c-KIT forms a bridge to Gab2 [17]–[19]. Phosphorylation of Gab2 by Src creates binding sites for PI3 kinase on Gab2. Since c-KIT is a pivotal player in hematopoiesis, its signaling has been well characterized in hematopoietic cells. Even though the importance of c-KIT in melanogenesis is recognized and a loss-of-function mutation of the receptor, or its downstream targets, can lead to developmental pigmentary diseases like piebaldism and Waardenburgs syndrome, the signaling cascades of c-KIT in melanocytes are not fully elucidated [20], [21].

Melanocytes are derived from the neural crest during embryogenesis. In order for these cells to fully differentiate into functional pigment producing melanocytes, these cells first have to migrate and colonize target tissues, including the skin and hair follicle. The program that enables such behavior is orchestrated by the c-KIT tyrosine kinase receptor and its target, the melanocyte master regulator Microphthalmia associated transcription factor (Mitf) [22]. Consequently, loss-of-function mutation of *Mitf* give rise to phenotypic defects in mice similar to that found in *c-KIT* and *SCF* mutant mice in that coat color is lacking due to absence of melanocytes and mutations in all three genes also affect mast cells [23], [24]. There are differences between *Mitf* mutations on the one hand and *c-KIT/SCF* mutations on the other hand in that *Mitf* affects eye and bone development whereas *c-KIT* and *SCF* do not; *c-KIT* and *SCF* have severe hematopoietic deficiencies which *Mitf* mutations do not exhibit, at least not to the same degree.

Mitf is a basic helix-loop-helix-leucine-zipper transcription factor that is not only essential for melanogenesis and melanocyte function, but is also involved in bone and mast cell development [25]–[28]. Mitf regulates a wide range of genes important for melanocyte and melanoma proliferation, survival, differentiation, apoptosis and cell cycle arrest (for review see [29]). The expression of the survival factor *CDK2* is maintained by Mitf in melanocytes and melanoma and the *BCL2* gene was found to be transcribed and upregulated by Mitf under SCF stimulation [30], [31]. In addition to cell survival, Mitf regulates *CDKN1A* and *CDKN2A* expression to inhibit cell growth and initiate differentiation [32], [33].

To date, two mechanisms are known by which c-KIT regulates the activity of Mitf. First, c-KIT phosphorylation activates the Ras-Erk pathway where Erk2 directly phosphorylates S73 of Mitf. As a result, the transactivation activity of Mitf is increased and the half-life of the protein is decreased as a consequence of ubiquitin-dependent degradation [34], [35]. Second, Erk activation leads to activation of the serine/threonine kinase p90 Rsk-1 that phosphorylates Mitf at S409. Un-phosphorylated mutants at S73 and S409 of Mitf render the transcription factor more stable and transcriptionally less active [34], [36]. At this point, however, it is not clear if S73 and S409 are the only phospho-acceptor sites in Mitf and, in fact, several pieces of evidence suggest that they are not. First, the Mitf protein has been shown to be phosphorylated on a number of serine residues, in addition to S73, and S409. These include S298 and S307 (reviewed in [29]). Second, genetic analysis of the role of signaling to Mitf during normal melanocyte development using knock-in and BAC transgene rescue strategies indicate that the S73 and S409 do not play a role during normal melanocyte development in mice [37], [38]. Clearly, the signaling to Mitf needs to be further characterized, both with respect to actual phosphoacceptor sites, and with respect to the signaling mechanisms involved to be able to understand the precise mechanism of regulation of Mitf activity.

In this report we have used a series of c-KIT mutants and pharmacological inhibitors to identify the signaling pathways radiating from c-KIT to Mitf in human embryonic kidney (HEK) 293T cells and in Melan-A melanocytes. We show that the tyrosine-to- phenylalanine mutations Y568F, Y570F and Y721F in c-KIT prevent activation of Mitf during SCF stimulation. In contrast, the Y703F/Y936F c-KIT double mutant does not block SCF-induced activation of Mitf. In addition, by using selective inhibitors against Src, Mek 1 and 2, p38, PI3 kinase and Akt, we show that the corresponding downstream signaling pathways are involved in the activation of Mitf. Finally, our transfection model revealed that the different c-KIT mutants together with wild type (wt) Mitf yielded a lower degree of SCF-induced proliferation compared to wild type c-KIT expressing HEK293T cells.

## Materials and Methods

### Cell culture

HEK293T cells (Thermo Scientific Open Biosystems) were cultured in Dulbecco's modified Eagle's medium and supplemented with 10% fetal bovine serum and 100 units/ml penicillin- streptomycin. Mouse melanocyte Melan-A cells were cultured according to recommended protocol (http://www.sgul.ac.uk/depts/anatomy/pages/dcbm&m.htm). The Melan-A cell line was a kind gift from Dr. Bennett.

BaF3 pro-B cells were maintained in RPMI-1640 medium supplemented with 100 units/ml penicillin- streptomycin, 10% heat inactivated fetal bovine serum and 10 ng/ml of IL-3.

### Plasmids

QuikChange mutagenesis kit (Stratagene) was used to generate tyrosine-to-phenylalanine substitution mutants of c-KIT contained in pcDNA3 vector. P3XFLAG-CMV-14 (Sigma Aldrich) was used as a vector backbone for the Mitf construct.

### Transfection

Turbofect transfection reagent (Fermentas) was used for all the experiments in this study according to manufacturer's protocol.

### Ligand stimulation and inhibitor treatment

SCF (ORF Genetics) was used for cell culture stimulation in a final concentration of 100 ng/ml. In the inhibitor experiments, 10 µM of SU6656 (CalBiochem), U0126 (Promega), LY294002 (Sigma Aldrich) or 3 µM of Akt IV (CalBiochem) was pre-incubated in cell culture medium for 30 minutes in 5% CO_2_, 37°C prior to SCF stimulation.

### Proliferation assay

HEK293T (4 million) cells were plated in 75 cm^2^ cell culture flasks (Nunc). One day after plating, cells were transfected with both c-KIT and/or Mitf plasmids and incubated for 8 hours in a cell culture incubator, after which the cells were washed 3 times with PBS and cultured in serum starved medium. Cells that were detached from culture flasks during the washing steps were collected and resuspended. SCF was added the day after the initial starvation procedure. Cells were trypsinized, resuspended in culture medium, stained with trypan blue and analyzed with Countess automated cell counter (Invitrogen) after 48 hours of SCF stimulation.

### Immunoblotting and immunoprecipitation

Cells were lysed for 15 minutes in cold lysis buffer containing 1% Triton X-100, 25 mM Tris, pH 7.5, 1 mM Na_3_VO_4_, 1% Trasylol, 1 mM phenylmethylsulfonyl fluoride, 5 mM EDTA and 25mM beta-glycerophosphate. The lysates were centrifuged at 17,000 X g for 20 minutes at 4°C. The FLAG-tagged Mitf protein was immunoprecipitated with monoclonal anti-FLAG M2 antibody (Sigma Aldrich). Human c-KIT was immunoprecipitated with the KitC1 antibody [39]. Mouse c-KIT was immunoprecipitated with the M-14 anti-c-KIT antibody (Santa Cruz, CA). After antibody addition, the cell lysate was incubated with rotation at 4°C for 1.5 hours. The immunoprecipitates were collected on protein G-Sepharose beads (GE Healthcare), washed three times in lysis buffer supplemented with above mentioned inhibitors. Protein elution was carried out by boiling 4 minutes in sample buffer (50 mM Tris-HCl pH 6.8, 2% SDS, 10% glycerol, 200 mM dithiothreitol, 12.5 mM EDTA and 0.02% bromophenol blue). After sample separation in SDS-PAGE with 8% continuous gel, proteins were electrotransferred to Immobilon P (Millipore) membranes using a semi-dry blotting system (BioRad). The membranes were blocked in 0.2% Tween 20 in PBS for 1 hour at room temperature. Primary and secondary horseradish peroxidase-conjugated antibody incubation was performed at room temperature for 1 hour. Prior to incubation with the secondary antibody the membranes were washed extensively with 0.5% Tween 20 in PBS. Finally, Immobilon Western chemiluminescent HRP substrate (Millipore) was used for protein detection.

### Mitf mobility shift quantitation

Mitf protein intensity was measured with ImageJ as outlined at the NIH website: http://rsbweb.nih.gov/ij/docs/menus/analyze.html. The intensity of the low molecular weight band was subtracted from the intensity of the heavy molecular weight band. According to this method a protein mobility shift seen as increased intensity of the heavy molecular weight protein band, produces a positive value. A resulting negative value indicates inactive state of Mitf. Each bar in the mobility shift quantitation charts in this study represents the average from three independent experiments. Error bars are reported as standard error of the mean.

### Statistical analysis

Each data set from Mitf mobility shift assays and cell proliferation experiments represents the mean for three and nine separate experiments, respectively. One way analysis of variance was used to analyze significant differences in cell proliferation assay. Significant results were further examined by a Newman- Keuls multiple comparison test. A *p*-value of <0.05 was considered significant. A 2-tail non parametric sign test was performed on Mitf mobility shift assay data, where the criterion for significance was set as *p*<0.05.

## Results

### Mitf is activated upon c-KIT stimulation

**Figure 1:**
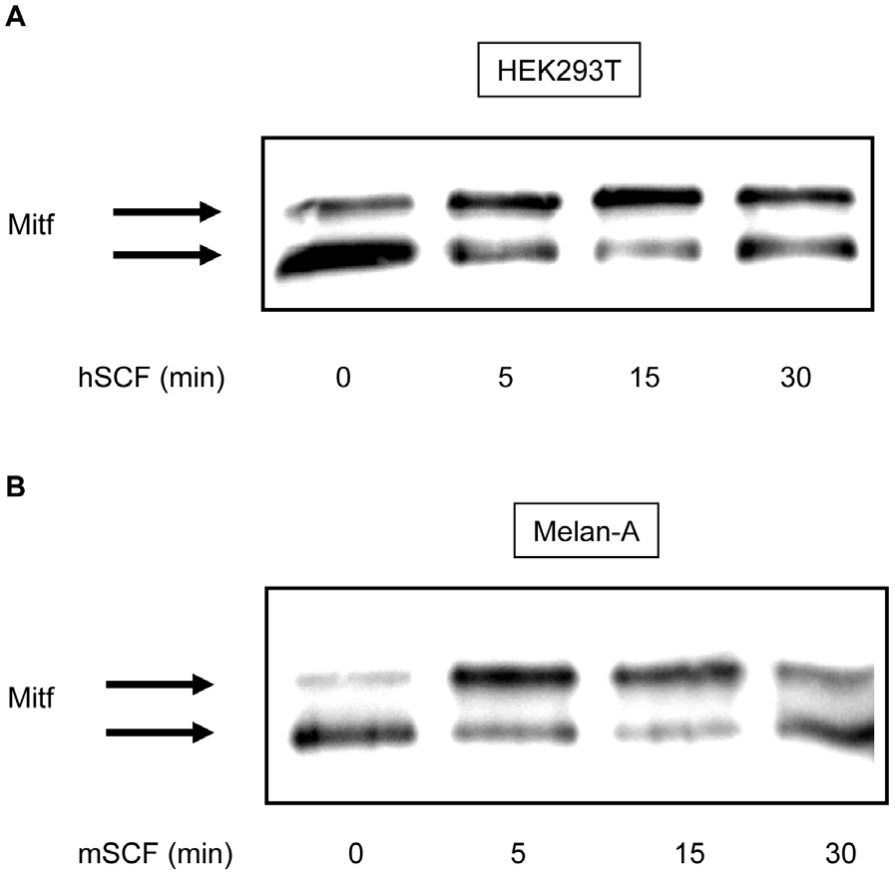
C-KIT activation induces a mobility shift of Mitf. (A) HEK293T cells transfected with c-KIT and Mitf and (B) Melan-A cells transfected with only Mitf displays mobility shift, where the upper band becomes more intense than the lower band, after 5–15 min of SCF (100 ng/ml) stimulation.

**Figure 2:**
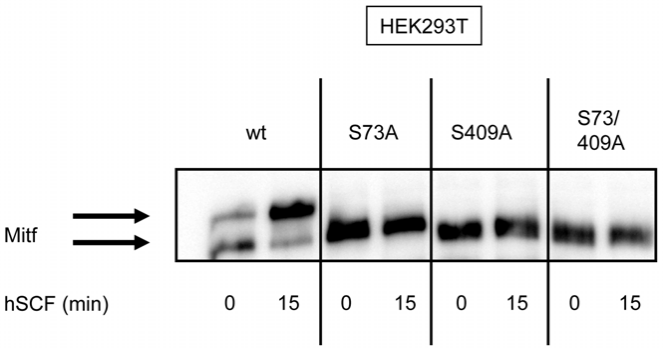
Mitf upper band is abolished by S73A, S409A and S73/409A substitution mutations. Transfection with wt Mitf in HEK293T cells shows a mobility shift after 15 minutes of SCF stimulation. In contrast, the S73A, S409A and S73/409A mutations of Mitf completely eliminate the heavy molecular weight band of Mitf, independent of hSCF treatment.

Ligand-stimulation of c-KIT post transcriptionally modifies Mitf through Erk-dependent phosphorylation of Mitf at S73 and S409. Addition of these phospho-groups can easily be detected by Western blotting where the modifications generate a mobility shift of the Mitf protein [34], [36]. Previous mobility shift studies were mostly performed with endogenous c-KIT and Mitf in melanocytes and melanoma cells. Because of this we needed to verify that our transfection model with HEK293T cells and mouse melanocyte Melan-A cells would produce equivalent results. Mitf in unstimulated state resolves as one heavy (upper) and one light (lower) molecular weight band with a higher intensity of the lower band. We transfected HEK293T cells with both c-KIT and Mitf whereas Melan- A cells were only transfected with Mitf. After 15 minutes of SCF stimulation in both HEK293T and Melan-A cells the band intensity of Mitf shifted resulting in increased intensity of the heavy band. (Fig. 1A & B). The intensity shift of Mitf was partly reversed back to basal state after 30 minutes of SCF stimulation. We further tested Mitf protein shift in HEK293T cells by generating serine to alanine substitution mutation of Mitf at position 73 and/or 409 and then transfecting these mutants or wt Mitf together with c-KIT. As expected, mobility shift of wt Mitf was detected after 15 minutes of SCF stimulation (Fig. 2). However, single substitution mutation of S73 or S409 and double mutation of S70/409A completely removed the upper band of the protein regardless of SCF stimulation. These results illustrate that SCF induced mobility shift of Mitf is dependent on S73 and S409. Collectively, our data from these cell lines are in agreement with previous reports, suggesting that our transfection model can be used for further signaling studies.

### C-KIT phosphorylation sites Y568, Y570 & Y721, but not Y703/963, are required for Mitf activation

**Figure 3:**
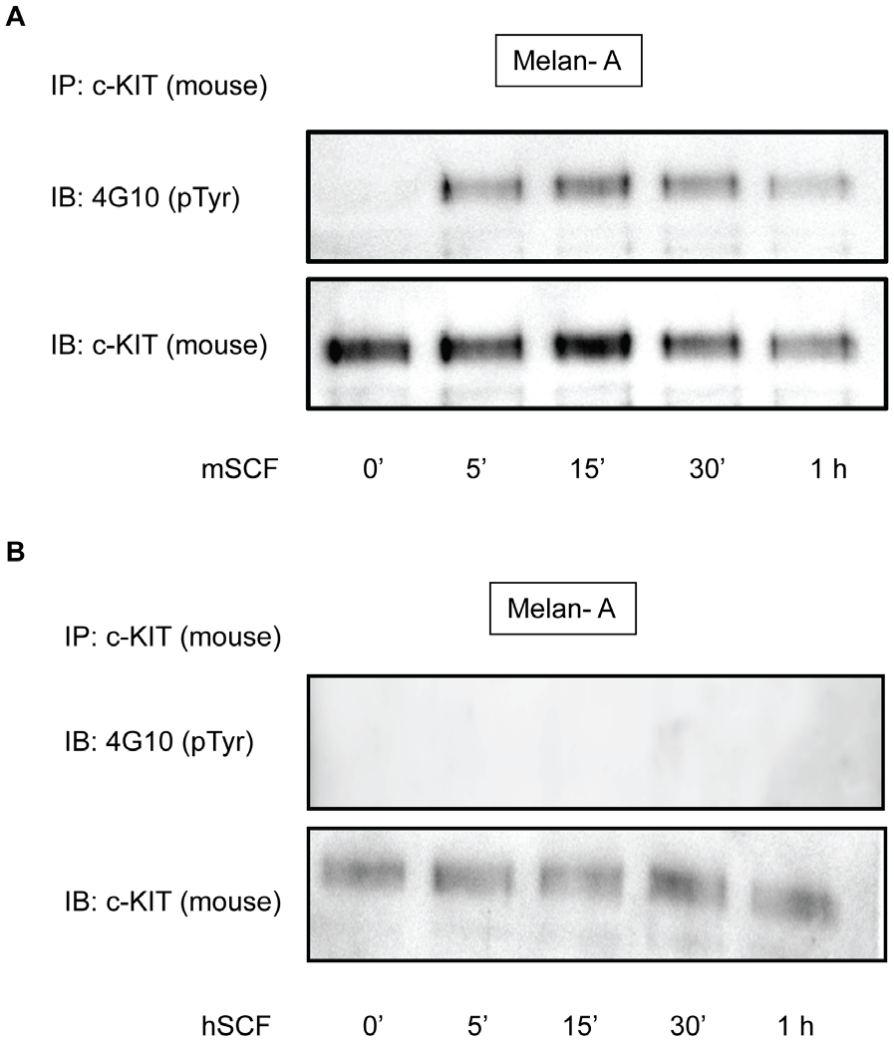
Human SCF (hSCF) does not activate mouse c-KIT. (A) Endogenous mouse c- kit immunoprecipitated from Melan-A cells stimulated with mouse SCF (mSCF) was autophosphorylated. (B) However, the addition of hSCF to the Melan-A cells did not activate endogenous mouse c-KIT. Immunoblotting with 4G10 antibody was used to detect general tyrosine phosphorylation to assess the c-KIT phosphorylation/ activation.

SCF binding to c-KIT causes auto-phosphorylation of various tyrosine residues in the intracellular domain of c-KIT. To pinpoint the involvement of the specific phospho-tyrosine residues involved in the SCF-induced activation of Mitf, we generated tyrosine-to-phenylalanine c-KIT mutants. In this experiment we used the HEK293T and mouse melanocyte Melan-A cell lines and transfected both c-KIT and Mitf into the cells. Because the Melan-A cells have endogenous expression of both wt Mitf and wt c-KIT, we needed to bypass the signals generated from wt mouse c-KIT in order to avoid interference with the transfected human c-KIT mutants. The activation of c-KIT can be detected by a general anti-phosphotyrosine monoclonal antibody (4G10 from Sigma Aldrich) after immunoprecipitation of the c-KIT protein. In the Melan-A cells, endogenous mouse c-KIT was phosphorylated after 5–15 min of mouse SCF (mSCF) stimulation (Fig. 3A). Furthermore, after 30 min to 1 hour of stimulation the level of endogenous c-KIT was decreased due to phosphorylation dependent internalization of the receptor. However, by using human SCF (hSCF) we were able to avoid the activation of the endogenous mouse c-KIT protein altogether (Fig. 3B).

**Figure 4:**
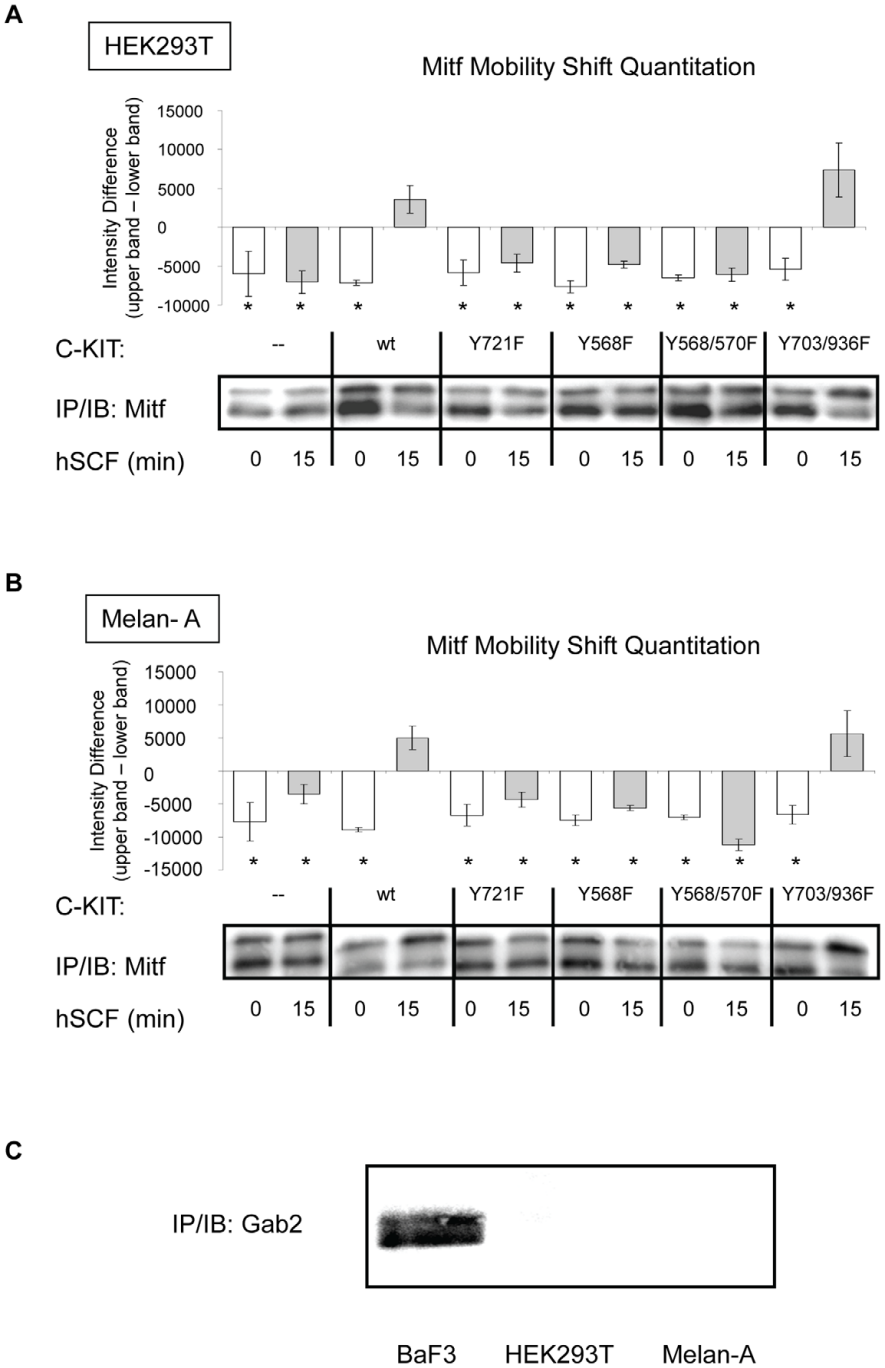
C-KIT phosphorylation mutants Y721F, Y568F and Y568F/Y570F are unable to activate Mitf. (A) HEK293T cells were transfected with wt or mutant forms of c-KIT and Mitf. C-KIT with mutated binding site for the PI3 kinase regulatory subunit p85 (Y721F), did not result in a Mitf band shift. C-KIT mutants Y568F and Y568F/Y570F, lacking the ability to activated Src, did not activate Mitf upon SCF stimulation. However, the c-KIT Y703F/Y936F mutant was able to mediate SCF-induced Mitf activation. (B) Mouse melanocytes Melan-A that were treated as above showed the same Mitf activation pattern. To statistically verify the results, Mitf mobility shift densitometric quantitation was performed on (A) HEK293T cells and (B) Melan-A cells. Each bar represents the mean ± SEM for at least three independent experiments. *Denotes significant difference from positive control *p = *0.00025. SFC treatment is indicated by gray bars. (C) Immunoprecipitation and subsequent immunoblotting of Gab2 protein reveal that neither HEK293T cells nor Melan- A melanocytes maintain an endogenous expression. In contrast, Gab2 protein is detected in the positive control BaF3 cell line.

Phosphorylation of c-KIT sites Y568 and Y570 (with Y568 being the primary activation site) allows docking and activation of Src family kinases leading to the activation of the Ras-Raf-Mek-Erk signaling cascade [15], [16]. The Erk signaling pathway as the activator of Mitf is well characterized [34], [36], [40], [41]. To determine whether the c-KIT phosphorylation sites Y568 and Y570 and potentially Src kinase are involved in the activation of Mitf we transfected c-KIT mutants Y568F and Y568/570F into both HEK293T and Melan-A cells together with Mitf. When stimulated with SCF these c-KIT mutants blocked the activation of Mitf as determined by reduced mobility shift of the protein (Fig. 4). Our results suggest that the c-KIT phosphorylation sites Y586 and Y570 are involved in the signaling to Mitf and that Src might play a role in this event.

The PI3 kinase survival pathway involving the serine/ threonine kinase Akt has been shown to protect melanocytes from apoptosis [42] and is also involved in the regulation of Mitf [43], [44]. The activation of the PI3 kinase pathway by c-KIT can be triggered through two alternative pathways [17]–[19]: first, through direct binding of p85 (subunit of PI3 kinase) to c-KIT phosphorylation site Y721. Second, c-KIT phosphorylation residues Y703 and Y936 can serve as binding sites for Grb2, allowing Grb2 to form a complex with Gab2, to which p85 can bind and interact. As a consequence of the interaction the PI3 kinase pathway is activated. In our transfection model, SCF stimulation of c-KIT Y721F mutant inhibited Mitf activation, indicating that phosphorylation of c-KIT at Y721 is needed in the activation of Mitf and that the PI3 kinase pathway might be involved (Fig. 4). However, despite impeded Grb2 binding sites, the c-KIT mutant Y703/936F was still able to cause a mobility shift of Mitf after the addition of SCF. Further examination showed that neither the HEK293T cells nor the Melan- A cells maintain an endogenous expression of Gab2 protein compared to the positive control pro-B BaF3 cells (Fig. 4C). Because c-KIT utilizes both direct (Y721) and indirect (Y703/936) binding of p85 to activate the PI3 kinase pathway, the lack of Gab2 in our cell lines favored the direct pathway, thus, rendering the Y703/936 c-KIT docking sites dispensable for the activation of Mitf.

In all, these observations illustrate that the activation of Mitf by c-KIT is dependent on functional direct docking sites for the PI3 kinase subunit p85 and Src kinase on c-KIT Y721 and Y568, respectively. Also, the data show that c-KIT phosphorylation sites Y703/936 are obsolete.

### C-KIT activates Mitf through the signaling proteins Src, Mek, PI3 kinase, Akt and p38

**Figure 5:**
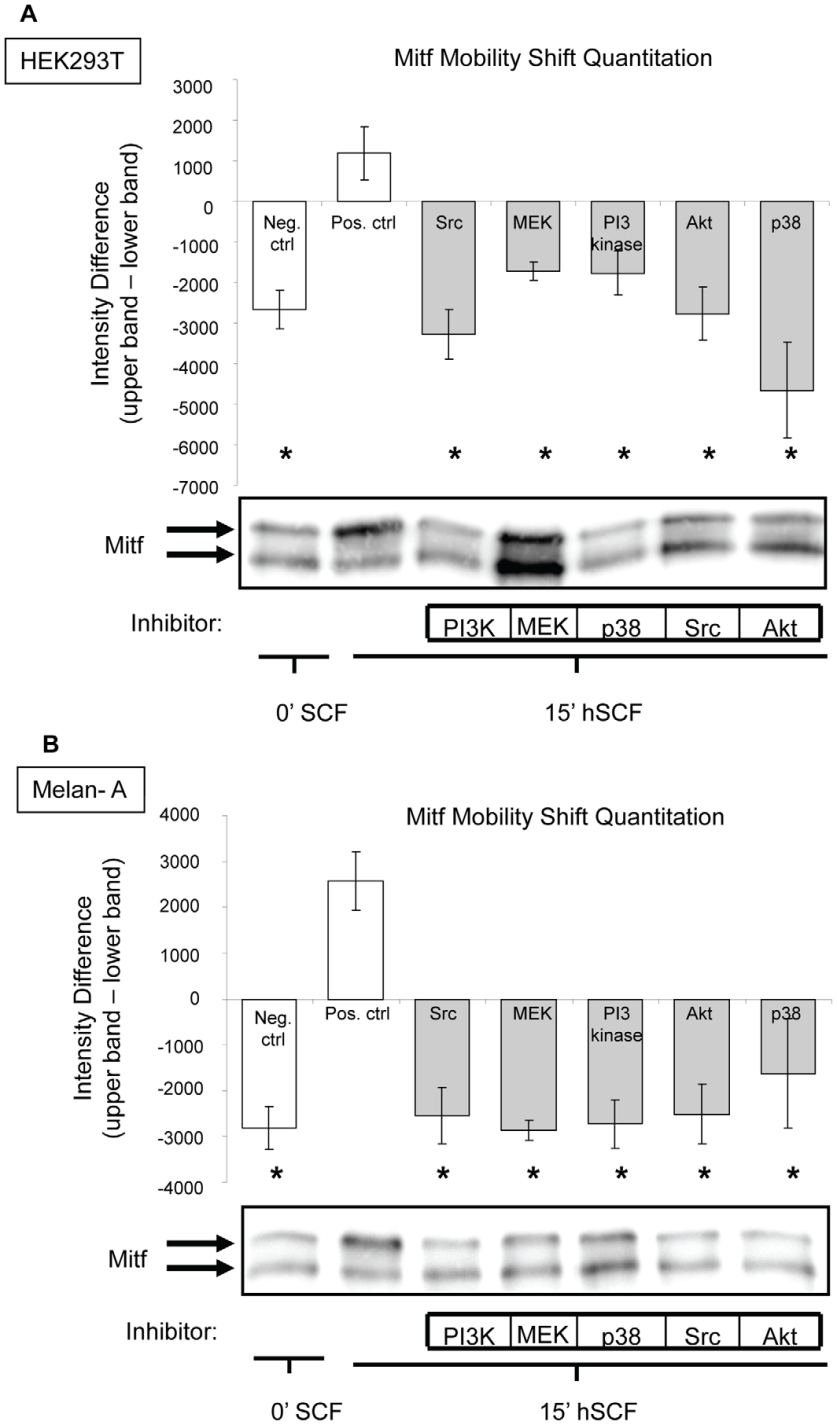
Inhibitors against Src kinase, Mek, PI3 kinase, Akt and p38 antagonize c-KIT mediated Mitf activation. Thirty minutes prior to SCF stimulation (A) HEK293T cells and (B) Melan-A cells were treated with Src family kinase inhibitor (SU6656), Mek inhibitor (U0126), PI3 kinase inhibitor (LY294002) or Akt inhibitor (Akt IV), respectively, all of which prevented c-KIT from activating Mitf. Densitometry analysis was done to statistically present Mitf mobility shift in (A) HEK293T cells and (B) Melan-A cells. Each bar represents the mean ± SEM for at least three independent experiments. *Denotes significant difference from positive control *p* = 0.00025. SFC treatment is indicated by gray bars.

The role of Src kinase in the activation of Mitf has not been investigated previously nor has the PI3 kinase-Akt pathway been studied in the context of c-KIT- induced Mitf activation. To investigate this, we treated cells with either Src family kinase inhibitor (SU6656), Mek inhibitor (U0126), PI3 kinase inhibitor (LY294002) or Akt inhibitor (Akt IV) for 30 minutes prior to SCF stimulation. The addition of SCF to untreated samples caused a mobility shift or activation of Mitf. However, pre-treatment with SU6656, U0126, LY294002 or Akt IV inhibited the activation of Mitf in all cases (Fig. 5). These observations suggest that Src kinases, Mek, PI3 kinase and Akt are all involved in SCF-induced Mitf phosphorylation.

The stress activated protein kinase p38 acts downstream of c-KIT and is known to be involved in the activation of Mitf gene expression in response to UV radiation [45]. In contrast, when human melanocytes are exposed to UV-B, p38 increases the expression of Mitf which in turn up-regulates the protein level of c-KIT via unknown mechanisms [46]. To test whether p38 is involved in SCF-induced Mitf activation, we employed the p38 inhibitor SU203580. Pre-treatment with SU203580 completely blocked SCF-induced Mitf mobility shift (Fig. 5), indicating that the signal from c-KIT to Mitf can also be mediated through p38.

Our results not only confirm that c-KIT activates Mitf through the Ras-Erk pathway as previously reported [34], [36], but show that this mechanism is Src kinase-dependent. Our inhibitor studies also demonstrate that upon SCF stimulation, c-KIT triggers the activation of Mitf through the stress activated p38 kinase and the survival PI3 kinase-Akt pathway.

### C-KIT and Mitf mediated cell proliferation

**Figure 6:**
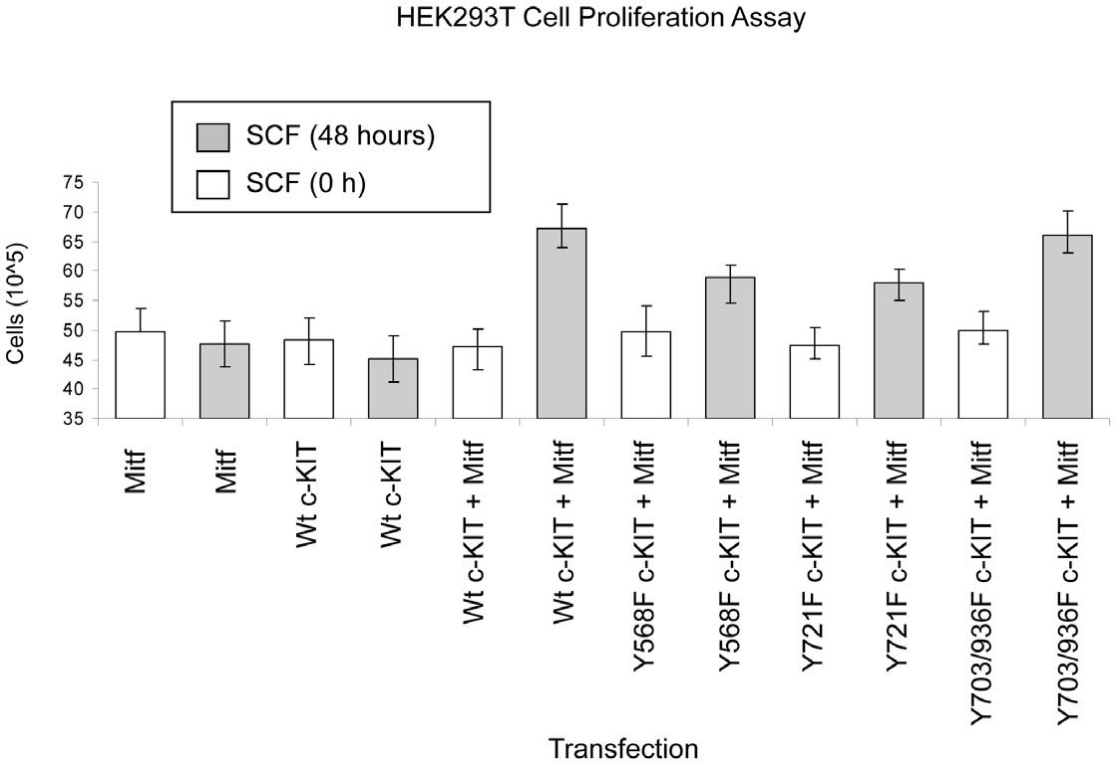
Effects of C-KIT and Mitf on cell proliferation. HEK293T cells transfected with either c-KIT or Mitf did not influence the level of cell proliferation regardless of SCF stimulation. However, when wt c-KIT or c-KIT Y703F/Y936F and Mitf were introduced, SCF treatment for 48 hours resulted in an increase in cell numbers. While the c-KIT mutants Y568F and Y721F were able to mediate a proliferative response to SCF stimulation, the level of increase was significantly (*p<*0.01) lower compared to wt c-KIT or c-KIT Y703F/Y936F. Each bar represents the average of nine independent experiments. Error bars signify 95% confidence interval.

Mitf activates transcription of a wide array of genes that regulate both cell survival and cell death [30], [41], [47], [48]. In melanocytes and melanoma the survival gene BCL2 is transcribed as a consequence of c-KIT- mediated Mitf activation [31]. To study the biological function resulting from the c-KIT and Mitf interaction in the HEK293T cell model we transfected this cell line with either wt c-KIT or mutant form of c-KIT together with wt Mitf. Treatment of serum-starved HEK293T cells with SCF for 48 hours in the presence of wt c-KIT and wt Mitf, promoted enhanced cell growth (Fig. 6). However, when the cells were transfected with the c-KIT mutants Y568F or Y721F together with wt Mitf, SCF stimulation yielded a significantly reduced level of cell growth compared to wt c-KIT. In contrast, the Y703F/Y936F double mutant of c-KIT that lacks functional Grb2 binding sites (that could bind to Gab2) did not significantly produce lower cell proliferation rate compared to wt c-KIT. This result can be explained by the fact that the HEK293T cells do not maintain an endogenous level of Gab2 protein (Fig. 4C). The addition of SCF for 48 hours to c-KIT Y568F, Y721F and Y703/936 mutants significantly (*p*<0.01, *p*<0.01 and *p*<0.001, respectively) augmented cell growth compared to respective 0 hour negative control. In addition, control cells transfected with only c-KIT or Mitf did not produce any proliferative effects upon SCF stimulation (Fig. 6).

Our results show that SCF-dependent cell proliferation is only achieved in the HEK293T cells when wt c-KIT and wt Mitf are present. Although, c-KIT mutants that lack functional Src and p85 binding sites can mediate ligand-induced cell proliferation, the response is severely impaired compared to the wild-type receptor.

## Discussion

The mechanisms behind c-KIT activation are well studied. When c-KIT is bound to its ligand, SCF, the receptor dimerizes, leading to auto-activation of the cytoplasmic tyrosine kinase domain [49]. Subsequent phosphorylation of additional tyrosine residues in c-KIT enables downstream signaling proteins to bind to the receptor. These events result in the activation of many different signaling pathways. In contrast, the c-KIT signaling mechanisms involved in the activation of Mitf in melanocytes are not fully understood. To date, the Ras-Erk pathway is the only known signaling cascade that has been shown to participate in the phosphorylation and activation of Mitf after SCF stimulation [34], [36]. Erk-2 has been shown to directly phosphorylate Mitf at S73 in both melanocytes and melanoma cells. Alternatively, c-KIT activation can also influence Erk-2 to activate the serine/threonine kinase p90 Rsk-1 to phosphorylate Mitf at S409. Phosphorylation of Mitf increases its transcriptional activity and decreases the protein stability. The biological outcome of this interaction in cell culture models is to promote melanocyte and melanoma cell survival. However, genetic analysis of the role of signaling to Mitf during normal melanocyte development suggests that neither S73 nor S409 are essential during normal melanocyte development in mice [37], [38]. Thus, the signaling from c-KIT to Mitf needs to be further characterized, especially with respect to the signaling mechanisms involved. As that both c-KIT and Mitf are essential factors in the development of melanocyte and melanoma and the role they play in related pathogenesis, it is of importance that the additional mechanisms and pathways that are controlled by c-KIT and regulate Mitf are identified. In this study we transfected HEK293T cells and mouse melanocytes, Melan-A, with c-KIT and Mitf to elucidate novel signal transduction pathways.

In melanoma, Src family of kinases has the ability to suppress differentiation and favor proliferation [50]. Src kinases are responsible for sustained Erk activation, whereby Mitf protein levels are diminished, resulting in abrogation of differentiation and pigment production. The c-KIT phosphorylation site Y568 located in the juxtamembrane region of the receptor can act as a docking site for Src family kinases. The association and phosphorylation of Src kinase by c-KIT leads to the activation of Src and phosphorylation of the adaptor protein Shc which in turn provides association sites for the Grb2/Sos complex and thereby activating the Ras-Erk pathway. Thus, a substitution mutation of c-KIT Y568 to phenylalanine would prevent the activation of the Ras-Erk pathway [14]. In our study, ligand stimulation of wt c-KIT caused a mobility shift of the Mitf protein. However, when the c-KIT mutant Y568F was presented with SCF, the Mitf mobility shift was absent. To further verify the involvement of Src kinase and Erk in the activation of Mitf, we complemented the experiment with an inhibitor study. The addition of the Src family kinase inhibitor (SU6656) and Mek (U0126) inhibitor reversed the effect of SCF-induced Mitf activation. Although, it is established that phosphorylation mediated mobility shift of Mitf is generated by the activation of the Ras-Erk Map kinase pathway, treatment with a general Mek inhibitor, U0126, did not completely eliminate the upper band of Mitf. Previous reports [34], [36] and our study (Fig. 2) demonstrate that the upper band is only present in wt Mitf and that S73A and S409A Mitf mutants exclusively resolve as a single lower band. Based on these observations one can draw the conclusion that phosphorylation of both S73 and S409 are required for the existence of the upper band. However, earlier studies [36] show that *in vitro* phosphatase treatment of SCF-stimulated Mitf only reversed SCF-induced mobility shift of the protein, leaving the upper band intact. Thus, it seems mobility shift of Mitf is the consequence of S73 and S409 phosphorylation and subsequent post translational modification that directly dictates the occurrence of the upper band. Since, *in vivo* inhibition of Mek did not completely abrogate the shift, this suggests the involvement of additional signaling pathways that might regulate the modification of Mitf.

The importance of the Ras-Erk pathway in the activation of Mitf is highlighted in this experiment, in agreement with previous studies. But, more importantly, we show that the interaction between Src kinase and c-KIT phosphorylation site Y568 is required for the activation of Mitf. The Ras-Erk pathway is known to be critical for cell proliferation and both c-KIT [14] and Mitf [41] have been shown to affect cell growth through this cascade. Correspondingly, our cell proliferation study revealed that in HEK293T cells transfected with Y568F c-KIT and Mitf, ligand-stimulated proliferation was greatly impaired compared to wt c-KIT.

One of the functions of Mitf is to indirectly protect the skin cells from UVB-induced DNA damage by transcribing genes that controls the production and transfer of melanin to adjacent keratinocytes. The p38 stress response pathway is triggered by UVB exposure in human melanocytes. As a result, the level of Mitf is increased and pigment producing genes are transcribed [45]. This stress pathway is also a signaling component downstream of c-KIT where the binding of Src kinase to mouse c-KIT Y567 (analogous to human c-KIT Y568) triggers the activation of p38 in bone marrow derived pro-B cells [16]. These observations prompted us to investigate whether there is a direct connection between c-KIT and Mitf through the p38 pathway. Indeed, by using a p38 inhibitor (SU203580) we show that SCF activation of Mitf is significantly reduced.

During progression of melanoma, expression of the scaffolding and adaptor protein Gab2 is often found to be amplified. The presence of Gab2 aggravates the invasiveness and mestastatic capabilities of melanoma [51]. Gab2 interacts with receptor tyrosine kinases including ErbB2 and c-KIT. This adaptor protein is activated through the binding to c-KIT phosphorylation sites Y703 and Y936 and potentiates the Ras-Erk and PI3 kinase-Akt pathways [17]–[19]. In our study the c-KIT mutant Y703F/Y936F, despite lacking the Gab2 binding sites, is still able to activate Mitf. This activity can be explained by the absence of Gab2 expression from both HEK293T and Melan-A cells. The fact that the expression of Gab2 either is low or absent in melanocytes [51] (Fig. 3E) together with the fact that it does not regulate Mitf in our study, suggest that the role of Gab2 in melanocytes is dispensable. Although, Gab2 might not be important in melanocytes it has been shown that Gab2 knock down melanoma cells exhibit decreased migration and invasion capabilities [51]. Thus, Gab2 and Grb2 signaling in melanoma cells does enhance oncogenic phenotype [52]; These Gab2-dependent phenotypes are reported to be mediated via the PI3 kinase-Akt signaling pathway.

The PI3 kinase-Akt survival pathway protects both melanocytes and melanoma from programmed cell death [42], [53]. Interestingly, inhibition of this signaling pathway increases the production of melanogenic enzymes through the stimulation of Mitf in mouse melanoma cells [54]. Clearly, the PI3 kinase-Akt pathway is involved in the regulation of melanocytic cells and Mitf is a target of this pathway. However, the participation of c-KIT in this process has never been studied. PI3 kinase can be activated by binding of its p85 subunit to c-KIT phosphorylation site Y721. Here we show that the c-KIT mutant Y721F transiently transfected in both HEK293T and Melan-A cells was unable to activate Mitf. In addition, inhibitors against PI3 kinase and Akt also blocked SCF-induced activation of Mitf. Thus, the signal transduction from c-KIT to Mitf seems to be relayed partly through the PI3 kinase-Akt pathway. In our study, blocking of this pathway by the Y721F mutant of c-KIT leads to decreased proliferation demonstrating the importance of the PI3-kinase pathway for Mitf- mediated HEK293T cell proliferation. On the other hand, this cascade might not be pivotal in melanocytes, since the disruption of PI3 kinase binding to c-KIT by an Y719F substitution- mutation of c-KIT (analogous to human c-KIT Y721) in mice did not produce any pigment defects [11], [12].

Our cell proliferation results point out another level of signaling complexity. As expected, the highest level of cell proliferation was achieved through the stimulation of wt c-KIT in the presence of Mitf. Even though SCF stimulation of the different mutants of c-KIT results in reduced Mitf- dependent cell growth, the mutants are still quite capable of promoting proliferation. This phenomenon can be partly explained by the involvement of other regulators in c-KIT signaling. The c-KIT phosphorylation sites Y568 and Y570 in the juxtamembrane domain serve as multifunctional docking sites. Mutation of one or the other leads to a loss of signalling through multiple pathways. The phosphorylated Y568 residue does not only act as binding site for Src family kinases [14], but also for the Csk homologous kinase [55] which is involved in negative regulation of the Src family kinase Lyn [56]. In addition, Y568 is the docking site for the protein tyrosine phosphatase SHP2. SHP2 is known mainly as a positive regulator of the Ras-Erk pathway. Another protein tyrosine phosphatase, SHP1, binds to Tyr570 [57]. Thus a number of positive as well as negative signals emanate from the juxtamembrane region. To complicate things further, Src family kinases are known to both positively regulates signalling through phosphorylation of Shc and subsequent activation of the Ras-Erk pathway[14] and through phosphorylation of GAB2 [19]. On the other hand, the ubiquitin E3-ligase Cbl is tyrosine phosphorylated by Src and involved in ubiquitination and degradation of c-KIT [58].

Tyrosine 703 and 936 are consensus Grb2 binding sites in that they have an asparagine residue located two amino acids downstream of the phosphorylated tyrosine residue. However, they do not seem to be directly involved in recruitment of Grb2-Sos to the receptor. Rather, Grb2 acts as an adapter recruiting either the negative regulator Cbl [59] to the receptor or to the scaffolding protein GAB2. Thus Y703 and Y936 can mediate both positive and negative signals by c-KIT.

Y721 is a consensus binding site for the p85 subunit of PI3-kinase. In cells not expressing GAB2, Y721 is essential for activation of PI3 kinase by c-KIT [17]. However, in cells expressing GAB2, activation of PI3-kinase is mediated both by direct binding of PI3-kinase to Tyr721 as well as indirect binding to tyrosine phosphorylated GAB2 [19].

To summarize, the signalling income of ligand-stimulation of c-KIT is very complex, with several pathways initiated by the receptor feeding into each other and influencing each other. Thus, when all these components come into play, an apparent loss of Mitf mobility shift caused by c-KIT mutants Y568F and Y721F does not necessarily translate into a phenotypic response such as complete lack of cell proliferation.

**Figure 7:**
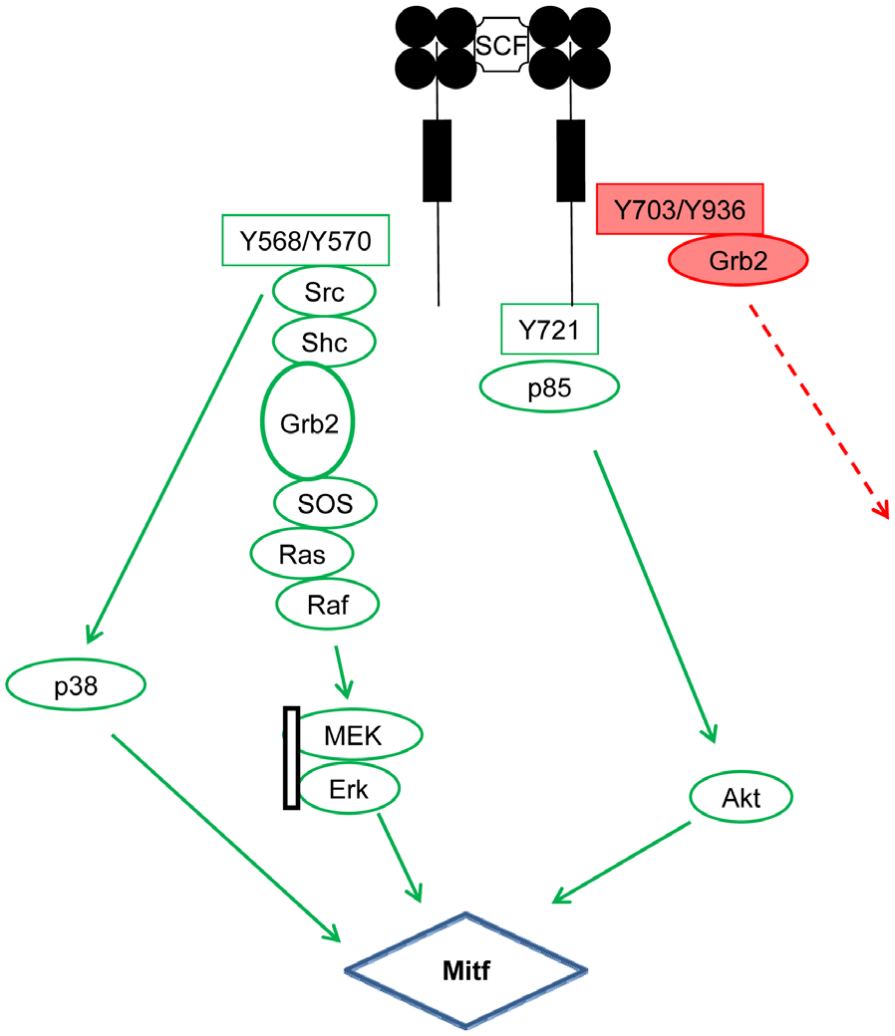
Proposed model for c-KIT mediated Mitf activation in HEK293T cells and mouse melanocytes, Melan- A. Phosphorylation of c-KIT Y568/Y570 recruits and Src kinase, triggering the activation of Ras/Raf/Mek/Erk and p38 kinase pathways which finally leads to Mitf activation. In contrast, phosphorylation of c-KIT Y721 results direct binding of p85 and subsequent Mitf activation through Akt. P85 can also indirectly interact with c-KIT through the binding of Gab 2. Since Gab 2 is absent in both HEK283T and Melan- A cells, the Y721 pathways is favored, rendering the Y703/Y936 dispensible for Mitf activation.

In summary, our data show that c-KIT regulates Mitf through the c-KIT phosphorylation sites Y568, Y570 and Y721 while Y703 and Y936 are dispensable (Fig. 7). We have identified the Src, p38, PI3 and Akt kinases to be involved in the regulation of Mitf. Furthermore, c-KIT mutants Y568F and Y721F lead to decreased Mitf-dependent cell proliferation compared to wt c-KIT. Collectively, our results suggest that the activation of Mitf by c-KIT is more complex than previously indicated. However, the involvement of the additional pathways we identified need to be further characterized to reveal their actual role in melanocyte and melanoma development.

## References

[1] Kasamatsu S, Hachiya A, Higuchi K, Ohuchi A, Kitahara T (2008). Production of the soluble form of KIT, s-KIT, abolishes stem cell factor-induced melanogenesis in human melanocytes.. J Invest Dermatol.

[2] Okayama Y, Kawakami T (2006). Development, Migration, and Survival of Mast Cells.. Immunol Res.

[3] Wehrle-Haller B (2003). The Role of Kit-Ligand in Melanocyte Development and Epidermal Homeostasis.. Pigment Cell Res.

[4] Vincent S, Segretain D, Nishikawa S, Nishikawa SI, Sage J (1998). Stage-specific expression of the Kit receptor and its ligand (KL) during male gametogenesis in the mouse:a Kit-KL interaction critical for meiosis.. Development.

[5] Chi P, Chen Y, Zhang L, Guo X, Wongvipat J (2010). ETV1 is a lineage survival factor that cooperates with KIT in gastrointestinal stromal tumours.. Nature.

[6] Alexeev V, Yoon K (2006). Distinctive Role of the cKit Receptor Tyrosine Kinase Signaling in Mammalian Melanocyte..

[7] Hachiya A, Sriwiriyanont P, Kobayashi T, Nagasawa A, Yoshida H (2009). Stem cell factor-KIT signalling plays a pivotal role in regulating pigmentation in mammalian hair.. J Pathol.

[8] Wang ZQ, Si L, Tang Q, Lin D, Fu Z (2009). Gain-of-function mutation of KIT ligand on melanin synthesis causes familial progressive hyperpigmentation.. Am J Hum Genet.

[9] Taylor ML, Dastych J, Sehgal D, Sundstrom M, Nilsson G (2001). The Kit-activating mutation D816V enhances stem cell factor--dependent chemotaxis.. Blood.

[10] Fritsche-Polanz R, Jordan JH, Feix A, Sperr WR, Sunder-Plassmann G (2001). Mutation analysis of C-KIT in patients with myelodysplastic syndromes without mastocytosis and cases of systemic mastocytosis.. Br J Haematol.

[11] Kissel H, Timokhina I, Hardy MP, Rothschild G, Tajima Y (2000). Point mutation in kit receptor tyrosine kinase reveals essential roles for kit signaling in spermatogenesis and oogenesis without affecting other kit responses.. EMBO J.

[12] Blume-Jensen P, Jiang G, Hyman R, Lee KF, O'Gorman S (2000). Kit/stem cell factor receptor-induced activation of phosphatidylinositol 3′-kinase is essential for male fertility.. Nat Genet.

[13] Ward SM, Sanders KM (2001). Physiology and pathophysiology of the interstitial cell of Cajal: from bench to bedside. I. Functional development and plasticity of interstitial cells of Cajal networks.. Am J Physiol Gastrointest Liver Physiol.

[14] Lennartsson J, Blume-Jensen P, Hermanson M, Pontén E, Carlberg M (1999). Phosphorylation of Shc by Src family kinases is necessary for stem cell factor receptor/c-kit mediated activation of the Ras/MAP kinase pathway and c-fos induction.. Oncogene.

[15] Roskoski R (2005). Signaling by Kit protein-tyrosine kinase- The stem cell factor receptor.. Biochem Biophys Res Commun.

[16] Ueda S, Mizuki M, Ikeda H, Tsujimura T, Matsumura I (2002). Critical roles of c-Kit tyrosine residues 567 and 719 in stem cell factor-induced chemotaxis: contribution of src family kinase and PI3-kinase on calcium mobilization and cell migration.. Blood.

[17] Serve H, Hsu YC, Besmer P (1994). Tyrosine residue 719 of the c-kit receptor is essential for binding of the P85 subunit of phosphatidylinositol (PI) 3-kinase and for c-kit-associated PI 3-kinase activity in COS-1 cells.. J Biol Chem.

[18] Lev S, Givol D, Yarden Y (1992). Interkinase domain of kit contains the binding site for phosphatidylinositol 3′ kinase.. Proc Natl Acad Sci U S A.

[19] Sun J, Pedersen M, Rönnstrand L (2008). Gab2 is involved in differential phosphoinositide 3-kinase signaling by two splice forms of c-Kit.. J Biol Chem.

[20] Tachibana M (1999). A cascade of genes related to Waardenburg syndrome.. J Investig Dermatol Symp Proc.

[21] Fleischman RA, Saltman DL, Stastny V, Zneimer S (1991). Deletion of the c-kit protooncogene in the human developmental defect piebald trait.. Proc Natl Acad Sci U S A.

[22] Hou L, Pavan WJ (2008). Transcriptional and signaling regulation in neural crest stem cell-derived melanocyte development: do all roads lead to Mitf?. Cell Res.

[23] Witte ON (1990). Steel locus defines new multipotent growth factor.. Cell.

[24] Russell ES (1979). Hereditary anemias of the mouse: a review for geneticists.. Adv Genet.

[25] Sharma SM, Bronisz A, Hu R, Patel K, Mansky KC (2007). MITF and PU.1 recruit p38 MAPK and NFATc1 to target genes during osteoclast differentiation.. J Biol Chem.

[26] Lu SY, Li M, Lin YL Mitf induction by RANKL is critical for osteoclastogenesis.. Mol Biol Cell.

[27] Nechushtan H, Razin E (2002). The function of MITF and associated proteins in mast cells.. Mol Immunol.

[28] Kitamura Y, Morii E, Jippo T, Ito A (2002). Regulation of mast cell phenotype by MITF.. Int Arch Allergy Immunol.

[29] Steingrimsson E, Copeland NG, Jenkins NA (2004). Melanocytes and the microphthalmia transcription factor network.. Annu Rev Genet.

[30] Du J, Widlund HR, Horstmann MA, Ramaswamy S, Ross K (2004). Critical role of CDK2 for melanoma growth linked to its melanocyte-specific transcriptional regulation by MITF.. Cancer Cell.

[31] McGill GG, Horstmann M, Widlund HR, Du J, Motyckova G (2002). Bcl2 regulation by the melanocyte master regulator Mitf modulates lineage survival and melanoma cell viability.. Cell.

[32] Carreira S, Goodall J, Aksan I, La Rocca SA, Galibert MD (2005). Mitf cooperates with Rb1 and activates p21Cip1 expression to regulate cell cycle progression.. Nature.

[33] Loercher AE, Tank EM, Delston RB, Harbour JW (2005). MITF links differentiation with cell cycle arrest in melanocytes by transcriptional activation of INK4A.. J Cell Biol.

[34] Wu M, Hemesath TJ, Takemoto CM, Horstmann MA, Wells AG (2000). c-Kit triggers dual phosphorylations, which couple activation and degradation of the essential melanocyte factor Mi.. Genes Dev.

[35] Xu W, Gong L, Haddad MM, Bischof O, Campisi J (2000). Regulation of microphthalmia-associated transcription factor MITF protein levels by association with the ubiquitin-conjugating enzyme hUBC9.. Exp Cell Res.

[36] Hemesath TJ, Price ER, Takemoto C, Badalian T, Fisher DE (1998). MAP kinase links the transcription factor Microphthalmia to c-Kit signalling in melanocytes.. Nature.

[37] Bauer GL, Praetorius C, Bergsteinsdottir K, Hallsson JH, Gisladottir BK (2009). The role of MITF phosphorylation sites during coat color and eye development in mice analyzed by bacterial artificial chromosome transgene rescue.. Genetics.

[38] Bismuth K, Skuntz S, Hallsson JH, Pak E, Dutra AS (2008). An unstable targeted allele of the mouse Mitf gene with a high somatic and germline reversion rate.. Genetics.

[39] Blume-Jensen P, Siegbahn A, Stabel S, Heldin CH, Rönnstrand L (1993). Increased Kit/SCF receptor induced mitogenicity but abolished cell motility after inhibition of protein kinase C.. Embo J.

[40] Molina DM, Grewal S, Bardwell L (2005). Characterization of an ERK-binding domain in microphthalmia-associated transcription factor and differential inhibition of ERK2-mediated substrate phosphorylation.. J Biol Chem.

[41] Wellbrock C, Rana S, Paterson H, Pickersgill H, Brummelkamp T (2008). Oncogenic BRAF regulates melanoma proliferation through the lineage specific factor MITF.. PLoS One.

[42] Oka M, Kageyama A, Fukunaga M, Bito T, Nagai H (2004). Phosphatidylinositol 3-kinase/Akt-dependent and -independent protection against apoptosis in normal human melanocytes.. J Invest Dermatol.

[43] Kim DS, Park SH, Kwon SB, Kwon NS, Park KC Sphingosylphosphorylcholine inhibits melanin synthesis via pertussis toxin-sensitive MITF degradation.. J Pharm Pharmacol.

[44] Lee JH, Jang JY, Park C, Kim BW, Choi YH Curcumin suppresses alpha-melanocyte stimulating hormone-stimulated melanogenesis in B16F10 cells.. Int J Mol Med.

[45] Saha B, Singh SK, Sarkar C, Bera R, Ratha J (2006). Activation of the Mitf promoter by lipid-stimulated activation of p38-stress signalling to CREB.. Pigment Cell Res.

[46] Mizutani Y, Hayashi N, Kawashima M, Imokawa GA, single UVB exposure increases the expression of functional KIT in human melanocytes by up-regulating MITF expression through the phosphorylation of p38/CREB.. Arch Dermatol Res.

[47] Carreira S, Goodall J, Denat L, Rodriguez M, Nuciforo P (2006). Mitf regulation of Dia1 controls melanoma proliferation and invasiveness.. Genes Dev.

[48] Dynek JN, Chan SM, Liu J, Zha J, Fairbrother WJ (2008). Microphthalmia-associated transcription factor is a critical transcriptional regulator of melanoma inhibitor of apoptosis in melanomas.. Cancer Res.

[49] Rönnstrand L (2004). Signal transduction via the stem cell factor receptor/c-Kit.. Cell Mol Life Sci.

[50] Wellbrock C, Weisser C, Geissinger E, Troppmair J, Schartl M (2002). Activation of p59(Fyn) leads to melanocyte dedifferentiation by influencing MKP-1-regulated mitogen-activated protein kinase signaling.. J Biol Chem.

[51] Horst B, Gruvberger-Saal SK, Hopkins BD, Bordone L, Yang Y (2009). Gab2-mediated signaling promotes melanoma metastasis.. Am J Pathol.

[52] Akavia UD, Litvin O, Kim J, Sanchez-Garcia F, Kotliar D An integrated approach to uncover drivers of cancer. Cell.

[53] Boisvert-Adamo K, Aplin AE (2006). B-RAF and PI-3 kinase signaling protect melanoma cells from anoikis.. Oncogene.

[54] Khaled M, Larribere L, Bille K, Ortonne JP, Ballotti R (2003). Microphthalmia associated transcription factor is a target of the phosphatidylinositol-3-kinase pathway.. J Invest Dermatol.

[55] Price DJ, Rivnay B, Fu Y, Jiang S, Avraham S (1997). Direct association of Csk homologous kinase (CHK) with the diphosphorylated site Tyr568/570 of the activated c-KIT in megakaryocytes.. J Biol Chem.

[56] Price DJ, Rivnay B, Avraham H (1999). CHK down-regulates SCF/KL-activated Lyn kinase activity in Mo7e megakaryocytic cells.. Biochem Biophys Res Commun.

[57] Kozlowski M, Larose L, Lee F, Le DM, Rottapel R (1998). SHP-1 binds and negatively modulates the c-Kit receptor by interaction with tyrosine 569 in the c-Kit juxtamembrane domain.. Mol Cell Biol.

[58] Masson K, Heiss E, Band H, Ronnstrand L (2006). Direct binding of Cbl to Tyr568 and Tyr936 of the stem cell factor receptor/c-Kit is required for ligand-induced ubiquitination, internalization and degradation.. Biochem J.

[59] Sun J, Pedersen M, Bengtsson S, Ronnstrand L (2007). Grb2 mediates negative regulation of stem cell factor receptor/c-Kit signaling by recruitment of Cbl.. Exp Cell Res.

